# The relationship of age, sex and prothrombin time related to the severity and mortality of COVID-19 patients with diabetes mellitus: a systematic review and meta analysis

**DOI:** 10.12688/f1000research.107398.2

**Published:** 2024-06-05

**Authors:** Audrey Fabianisa Mirza, Ceria Halim, Mutiara Indah Sari

**Affiliations:** 1 Faculty of Medicine, Universitas Sumatera Utara, Medan, Sumatera Utara, 20155, Indonesia; 2 Department of Biochemistry, Universitas Sumatera Utara, Medan, Sumatera Utara, 20155, Indonesia

**Keywords:** age, sex, prothrombine time, COVID-19, diabetes mellitus

## Abstract

**Background:**

SARS-CoV-2 first appeared in Wuhan, China, in December 2019. Looking at the prevalence data in the world and in Indonesia, the highest mortality rate due to COVID-19 involves age, gender and comorbidities such as diabetes mellitus. Severity of the condition also refers to coagulation abnormalities, such as abnormal prothrombin time values.

**Methods:**

This systematic review study and meta-analysis used online literature sourced from PubMed, Science Direct, EBSCO, Cochrane and Google Scholar. The literature used here is literature that has data on age, sex and prothrombin time of COVID-19 patients with diabetes mellitus whose quality is assessed by the NOS (Newcastle-Ottawa Scale) criteria and processing data using Review Manager 5.4.

**Results:**

Out of 8711 literatures that were traced from various search sources, there were 46 literatures that were included in this study. The results of the analysis on age showed the Standardized Mean Difference (SMD) value of 0.45 and P <0.0001 (95% CI: 0.23–0.68), the gender analysis showed an Odds Ratio (OR) value of 3.28 and P = 0.01 (95% CI: 1.26–8.52) and the prothrombin time analysis showed SMD values of 0.41 and P = 0.07 (95%CI = -0.03–0.85).

**Conclusion:**

Older and male COVID-19 patients have a higher risk of having diabetes compared to younger and female COVID-19 patients. As diabetes is a comorbidity in COVID-19, it can be concluded that old age and male sex are associated with a more severe disease.

## Introduction

SARS-CoV-2 first appeared in Wuhan, China, on December 31, 2019 and quickly spread throughout the world. As of April 13, 2021, a total of 136,291,755 confirmed cases of COVID-19 infection with 2,941,128 confirmed cases of death have been reported in 223 countries and territories worldwide. ^1^ In Indonesia, according to the National Development Planning Agency/Bappenas, the first confirmed case of COVID-19 was on March 2, 2020. On April 14, 2021, there were 1,577,526 positive confirmed cases and 42,782 for the number of deaths (2.7% of the national confirmed number). ^2^

Diabetes mellitus (DM) is an independent prognostic factor for COVID-19 patients. The survival rate of diabetic patients is lower, and the time from the on-set of the infection to death is shorter than that of non-diabetic patients. ^3^ The mechanism of expression of angiotensin-converting enzyme 2 (ACE2) is increased in lung and other tissues of DM patients. This upregulation is associated with chronic inflammation, activation of endothelial cells and insulin resistance which exacerbates the inflammatory response, in short, the clinical course and prognosis of COVID-19 in DM patients is significantly worse. ^4^

There is an increase in the number of cases and a greater risk of severe disease with age. ^5^ The increase in male mortality is related to the regulation of ACE2 and the body's immune system. ^6^ DM patients are in a prothrombotic state due to hyperglycemia and chronic hyperinsulinism. ^7^

Studies on COVID-19 associated with diabetes comorbid conditions have been studied by several researchers. However, the results obtained regarding age, gender and prothrombin time showed a lot of variability, so further exploration is needed to determine their association with diabetes on COVID-19. This systematic review and meta-analysis aims to examine the relationship between age, gender and prothrombin time on the severity and mortality of COVID-19 patients with DM as a comorbidity.

## Methods

### Data sources and search strategy

This research was conducted after obtaining approval from the Health Ethics Commission of Universitas Sumatera Utara (EC No.789/KEP/USU/2021). This study used online literature from PubMed, Science Direct, EBSCO, Cochrane and Google Scholar. The journals used were those which captured the data on COVID-19 patients having comorbid DM, accompanied by data on age, sex and prothrombin time values. The literature search was carried out according to PRISMA (Preferred Reporting Items for Systematic Reviews and Meta Analysis). The Checklist used in this meta-analysis was the PRISMA 2009 checklist.

This research was conducted in Medan, North Sumatra and was conducted between July–October 2021. Literature search was performed on the databases with the keywords “Age” AND (“Sex” OR “Gender”) AND (“Prothrombine Time” OR “PT”) AND (“COVID -19” OR “SARS CoV-2”) AND (“Diabetes Mellitus” OR “DM”) for articles published from 2019 to 2021. Filters on each database were utilized to aid the literature search: text availability (free full text), article attribute (associated data), article type (clinical trial and randomized control trial), and publication date (five years) for PubMed; years (2019, 2020, 2021), article type (research article, case reports, and data articles), publication title (International Journal of Infectious Diseases, the Lancet Infectious Diseases, the Brazilian Journal of Infectious Diseases), subject areas (medicine and dentistry, immunology and microbiology, nursing and health professions), language (English), and access type (open access and open archive) for Science Direct; date (custom range 2019-2021), topic (infectious diseases, endocrine & metabolic), Cochrane protocols, and Cochrane trials for Cochrane; custom range (2019-2021), sort by relevance, and any type for Google Scholar; and full text availability for Ebsco.

### Inclusion criteria

All retrospective studies (cross-sectional, cohort and case-control) that had data on patients’ age, sex and prothrombin time values who had been hospitalized either on the ward or in the ICU were considered eligible for this study. Eligible studies compared data on age in DM and non-DM patients, gender in DM patients and prothrombin time values in DM and non-DM patients.

### Exclusion criteria

All duplications were removed at the initial screening, followed by a second screening in which articles that did not meet the inclusion criteria were removed, such as review articles, systematic reviews, meta-analytical studies, comments, letters, animal studies and studies that were not in Indonesian or English.

### Journal quality review

The quality of the literature used in this study was determined based on criteria of NOS (The Newcastle Ottawa Scale) and for the selection a score of 7–9 (high quality study) was used.

### Method of collecting data

All relevant data was collected using data collection standards that had been set by two reviewers (AFM and MIS). The data taken for the age variable was the Mean and Standard Deviation (SD) of COVID-19 patients with DM and non-DM, the gender variable noted the Odds Ratio (OR) and Standard Error (SE) data from COVID-19 patients with DM, the variables taken for prothrombin time were the median and Interquartile range (IQR) which are converted into the Mean and SD of COVID-19 patients with DM and non-DM. Data was obtained from patients who had COVID-19 confirmed through reverse-transcriptase polymerase chain reaction (RT-PCR). These patients were interviewed regarding congenital diseases and blood tests was used to determine whether they had DM before being admitted to the hospital.

### Data analysis

This study used Review Manager 5.4 software (The Cochrane Collaboration, Oxford, UK) (RevMan, RRID:SCR_003581). ^55^ Standardized Mean Difference (SMD) and OR were used to analyze the variables in this study. The Confidence Interval (CI) was set at 95%. P value less than 0.05 indicated statistically significant data. Chi Square test was used to assess the heterogeneity of statistical data with the symbol I ^2^. If the I ^2^ test was worth more than 50%, it indicated that there was heterogeneity between studies and the study was conducted using a random effects model. If the I ^2^ test was less than 50%, it indicated that there was homogeneity between studies, the research was carried out using a fixed effects model. To reduce heterogeneity, studies which method may lead to clinical diversity that conflict with the rest of the studies are excluded. Data input was rechecked by all reviewers to ensure that they are correct.

## Results

### Study characteristics

**Figure 1.**
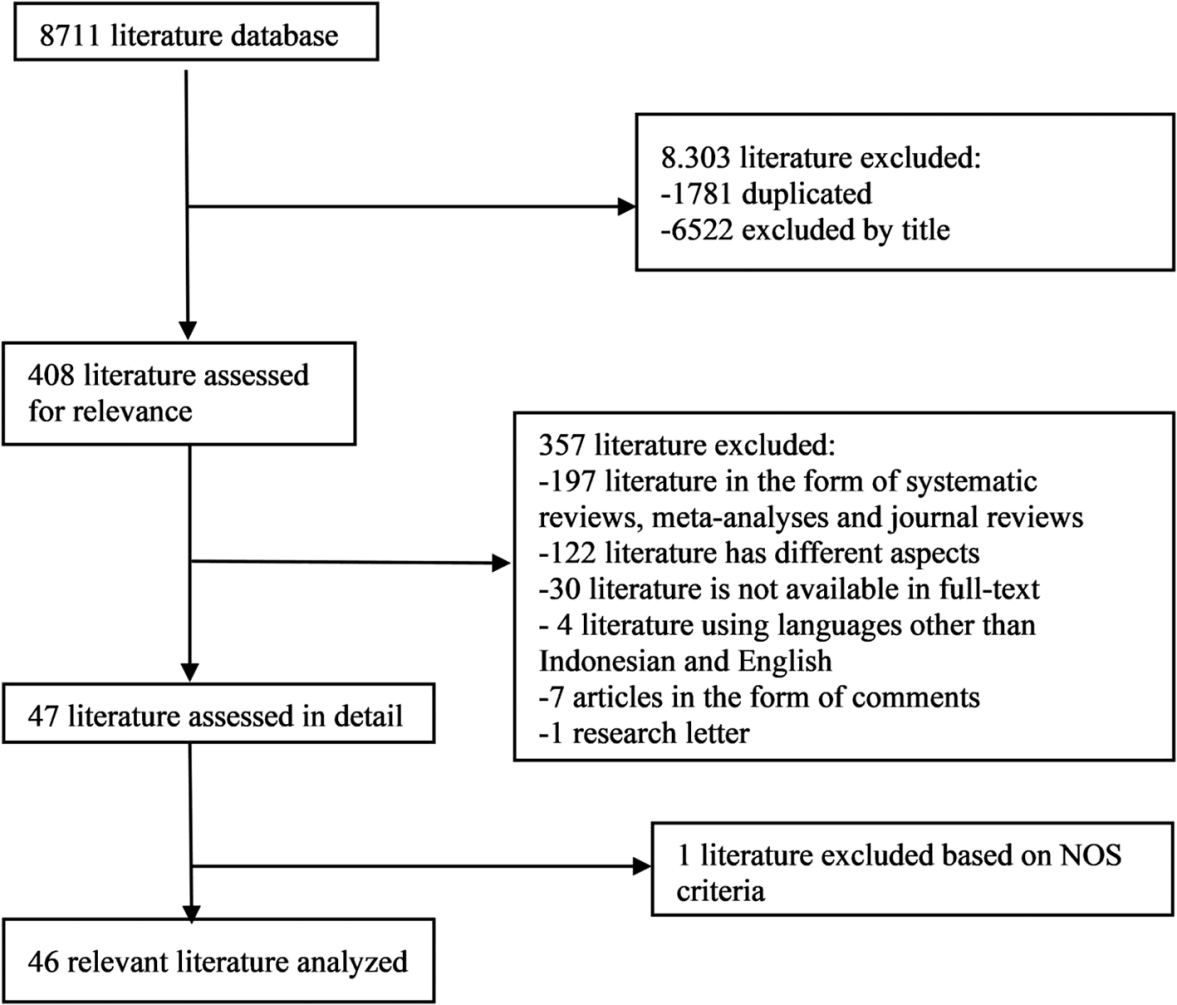
Literature Search.

In the initial search, we found 8711 articles which can be seen in Figure 1. The final results after selection got a total of 46 articles that were included in this meta-analysis study.

This meta-analysis study included literature examining two groups, namely COVID-19 patients (controls) and COVID-19 patients with DM. Data from both groups were taken from medical records of patients who were treated either in the ward or in the Intensive Care Unit (ICU). Characteristics of patients based on the study literature are seen in Table 1.

**Table 1.** Study characteristics.

Research by	Year	Location	Number of patients
Acharya *et al.* ^16^	2020	Korea	324
Alkundi *et al.* ^19^	2020	England	232
Ortega *et al.* ^39^	2021	Spain	2,069
Alshukry *et al.* ^20^	2021	Kuwait	417
Chen (a) *et al.* ^7^	2020	Wuhan, China	1,105
Chung *et al.* ^24^	2020	South Korea	117
Dennis *et al.* ^25^	2021	England	19,256
Pazoki *et al.* ^40^	2021	Iran	574
Elemam *et al.* ^8^	2021	United Arab Emirates	350
Jing Liang *et al.* ^28^	2020	Wuhan, China	211
Kim *et al.* ^29^	2020	South Korea	1,082
Koh *et al.* ^30^	2021	Singapore	1,042
Chen (c) *et al.* ^23^	2020	Wuhan, China	904
Pazoki *et al.* ^40^	2021	Iran	574
Wang *et al.* ^46^	2020	Wuhan, China	663
Liu (c) *et al.* ^34^	2020	Wuhan, China	192
Chen (b) *et al.* ^22^	2020	Hubei, China	208
Shang *et al.* ^44^	2021	Wuhan, China	584
Zhang (a) *et al.* ^51^	2020	Wuhan, China	258
Zhang *et al.* ^50^	2021	Wuhan, China	131
Leon-Abarca *et al.* ^31^	2021	Mexico	1,280,806
Dozio *et al.* ^26^	2020	Italy	33
Liu (a) *et al.* ^32^	2020	Chengdu, China	95
Liu (b) *et al.* ^33^	2020	Wuhan, China	268
Liu (d) *et al.* ^35^	2020	Wuhan, China	934
Makker *et al.* ^36^	2021	France	843
Mansour *et al.* ^37^	2020	Iran	353
Orioli *et al.* ^38^	2021	Belgium	192
Ozder *et al.* ^13^	2020	Turkey	640
Raghavan *et al.* ^41^	2021	India	845
Ricchio *et al.* ^42^	2021	Italy	61
Seiglie *et al.* ^43^	2020	America	450
Soliman *et al.* ^11^	2020	Qatar	299
Sticchi *et al.* ^14^	2021	Italy	1,656
Wu (a) *et al.* ^47^	2020	Wuxi, China	63
Wu (b) *et al.* ^48^	2020	Jiangsu, China	2,455
Xu *et al.* ^49^	2020	Wuhan, China	61
Zhang (b) *et al.* ^52^	2020	Wuhan, China	166
Zheng *et al.* ^53^	2021	Wuhan, China	71
Akbariqomi *et al.* ^17^	2020	Iran	595
Bhandari *et al.* ^21^	2021	Iran	53
Dyusupova *et al.* ^27^	2021	Kazakhstan	1,961
Huang *et al.* ^10^	2020	Wuhan, China	1,443
Li *et al.* ^12^	2020	Wuhan, China	199
Shi *et al.* ^45^	2020	Wuhan, China	306
Yan *et al.* ^18^	2020	Wuhan, China	193

Based on the entire literature that was included as many as 46 researched in 2020–2021, the most research was carried out in 2021 in as many as 28 studies. The country that researched the most literature in this meta-analysis was China, which was the initial location for the spread of COVID-19 as per as many as 21 literatures. Some studies have a small sample size, but the samples studied are COVID-19 patients who have been hospitalized and have moderate-severe symptoms so that they represent a patient population with a high risk of severity and mortality.

### The relationship between age and diabetes mellitus in COVID-19 patients

The literature that was included in the age distribution associated with the incidence of COVID-19 in DM and non-DM was 31 literatures. Among them were 3 literature cross-sectional research designs, 9 literature cohorts and 19 case-control literatures. Characteristics of age in patients based on the study literature can be seen in Table 2.

**Table 2.** Age studies in diabetes mellitus (DM) patients with COVID-19 and non-diabetic patients with COVID-19.

Journal	Research design	NOS Score	Age in DM (Mean ± SD)	Age in non-DM (Mean ± SD)
Acharya *et al*. ^16^	Cross-sectional	9	69.8 ± 13.5	51.9 ± 21.4
Alkundi *et al*. ^19^	Cross-sectional	8	71.4 ± 13.1	69.9 ± 17.1
Ortega *et al*. ^39^	Cross-sectional	8	71.7 ± 11.9	66.6 ± 16.3
Alshukry *et al.* ^20^	Cohort	9	56.4 ± 11.64	39.5 ± 16.59
Chen (a) *et al*. ^7^	Cohort	9	63.4 ± 12.8	55.3 ± 14.5
Chung *et al*. ^24^	Cohort	8	66.3 ± 8.9	53.5 ± 17.9
Dennis *et al*. ^25^	Cohort	9	67.0 ± 14.1	66.0 ± 17.4
Pazoki *et al*. ^40^	Cohort	9	65.0 ± 12.1	53.2 ± 16.7
Elemam *et al*. ^8^	Cohort	9	53.73 ± 12.79	44.64 ± 14.38
Jing Liang *et al*. ^28^	Cohort	7	62.4 ± 7.7	63.3 ± 8.3
Kim *et al*. ^29^	Cohort	9	68.3 ± 11.9	56.5 ± 18.0
Koh *et al*. ^30^	Cohort	9	48.0 ± 13.0	36.0 ± 10.0
Leon-Abarca *et al*. ^31^	Case-control	7	57.4 ± 13.4	41.8 ± 14.7
Dozio *et al*. ^26^	Case-control	8	72.6 ± 15.8	55.6 ± 22.5
Liu (a) *et al*. ^32^	Case-control	8	59.36 ± 12.31	58.0 ± 19.24
Liu (b) *et al*. ^33^	Case-control	8	65.54 ± 11.28	64.82 ± 10.98
Liu (d) *et al*. ^35^	Case-control	8	64.5 ± 10.0	61.6 ± 14.5
Makker *et al*. ^36^	Case-control	8	65.36 ± 13.96	58.6 ± 17.53
Mansour *et al*. ^37^	Case-control	8	63.66 ± 13.32	60.76 ± 17.56
Orioli *et al*. ^38^	Case-control	8	67.0 ± 14.0	67.0 ± 14.0
Ozder *et al*. ^13^	Case-control	7	57.0 ± 11.03	58.02 ± 12.16
Raghavan *et al*. ^41^	Case-control	8	60.0 ± 13.0	51.0 ± 17.0
Ricchio *et al*. ^42^	Case-control	8	81.0 ± 16.0	75.0 ± 15.0
Seiglie *et al*. ^43^	Case-control	8	66.7 ± 14.2	61.1 ± 18.8
Soliman *et al*. ^11^	Case-control	8	52.1 ± 12.67	36.22 ± 11.43
Sticchi *et al*. ^14^	Case-control	8	70.9 ± 11.0	66.3 ± 14.0
Wu (a) *et al*. ^47^	Case-control	7	47.98 ± 15.11	51.0 ± 12.6
Wu (b) *et al*. ^48^	Case-control	8	52.55 ± 13.7	47.98 ± 15.11
Xu *et al*. ^49^	Case-control	8	65.6 ± 11.11	62.96 ± 10.71
Zhang (b) *et al*. ^52^	Case-control	7	65.6 ± 11.4	59.4 ± 16.0

NOS: Newcastle-Ottawa Scale.

**Figure 2.**
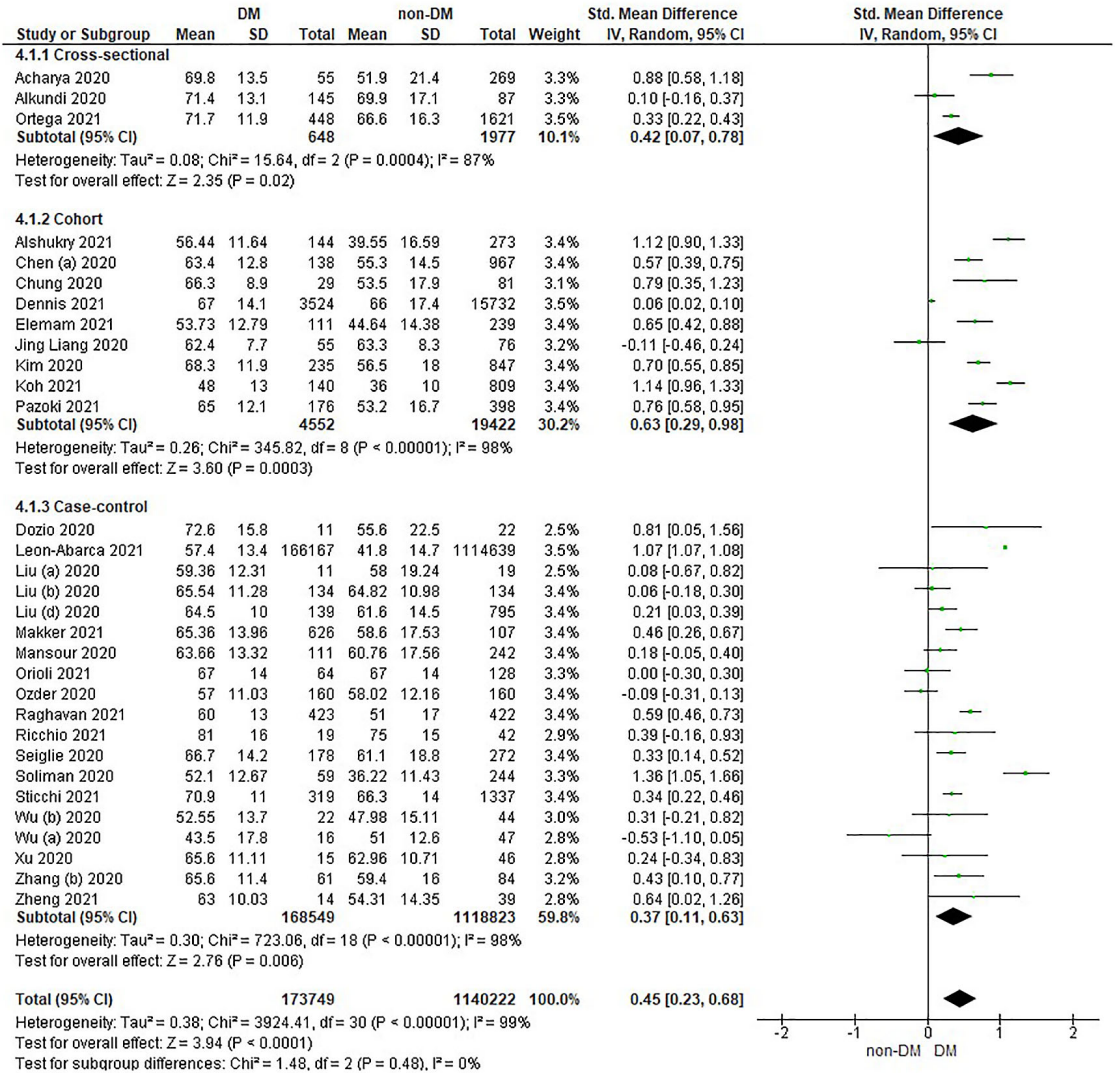
Forest plot of the relationship of age in diabetes mellitus to COVID-19 and non-diabetes mellitus to COVID-19.

Based on Table 2, the age distribution of the incidence of COVID-19 with DM compared to non-DM, almost all studies have data on patients with DM having an older age. Forest plot analysis of the relationship between age and the incidence of COVID with DM and non-DM can be seen in Figure 2.

The results of the literature analysis in the sub-group of cross-sectional study designs to see the comparison of age in COVID-19 patients with DM and non-DM resulted in I ^2^ = 87% indicating heterogeneity between studies. Subtotal SMD was 0.42 (95%CI = 0.07–0.78; P = 0.02) which indicates that the result was significant because P < 0.05 and the diamond did not touch the vertical line.

The results of the analysis in the cohort study design sub-group to see the comparison of age in COVID-19 patients with DM and non-DM resulted in I ^2^ = 98% which indicates heterogeneity between studies. Subtotal SMD was 0.63 (95%CI = 0.29–0.98; P = 0.0003) which is that the result was significant, because P < 0.05 and the diamond did not touch the vertical line.

The results of the analysis in the case-control study design sub-group to see the comparison of age in COVID-19 patients with DM and non-DM resulted in I ^2^ = 98% which indicates heterogeneity between studies. Subtotal SMD was 0.37 (95%CI = 0.11–0.63; P = 0.006) which indicates that the result was significant because P < 0.05 and the diamond did not touch the vertical line.

The results of the literature analysis to see the comparison of age in COVID-19 patients with DM and non-DM as a whole resulted in a value of I ^2^ = 99% which indicated heterogeneity between studies, so the random effects model was used. Total SMD 0.45 (95%CI = 0.23–0.68; P < 0.0001) with a population confidence interval of 0.23 to 0.68 (P < 0.0001) indicating there is a significant result because P < 0.05. In addition, the diamond did not touch the vertical line thus proving that COVID-19 patients with DM have an older age compared to COVID-19 patients without comorbid DM who are hospitalized.

### The relationship between sex and diabetes mellitus in COVID-19 patients

The literature that was included in the sex distribution was associated with the incidence of COVID-19 in DM as many as 5 literatures. Among them were 2 literature cross-sectional research designs and 3 literature cohorts. Gender characteristics of patients based on the study literature can be seen in Table 3 below.

**Table 3.** Study of gender in diabetes mellitus patients with COVID-19.

Journal	Research design	NOS Score	Male vs. Female (OR, 95%CI)
Acharya *et al*. ^16^	Cross-sectional	9	0.948 (0.13–6.92)
Ortega *et al*. ^39^	Cross-sectional	8	2.14 (1.014–4.5)
Chen (c) *et al*. ^23^	Cohort	9	0.36 (0.17– 0.77)
Pazoki *et al*. ^40^	Cohort	9	1.49 (0.77–2.87)
Wang *et al.* ^46^	Cohort	9	2.81 (0.90– 9.21)

NOS: Newcastle-Ottawa Scale.

**Figure 3.**
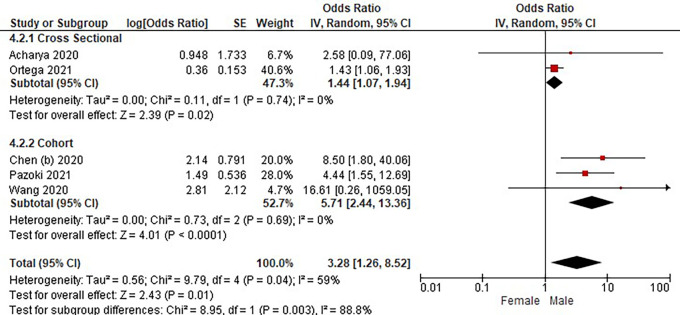
Forest plot of sex relationship in diabetes mellitus patients with COVID-19.

Based on Table 3, the sex distribution of the incidence of COVID-19 with DM, overall data on the OR value shows that male patients are more at risk of exposure to the disease than women. The forest plot analysis of the sex relationship with the incidence of COVID with DM can be seen in Figure 3.

Based on the results of the picture of the size of the square on the forest plot, the research by Ortega *et al.* (2021) has the largest square proportional to the greater weight value because it has a larger sample than other studies and has more influence on the results of this forest plot.

The results of the analysis on the sub-group cross-sectional study design to see the sex comparison in COVID-19 patients with DM resulted in I ^2^ = 0% which indicated the absence of heterogeneity between studies. Subtotal OR 1.44 (95%CI = 1.07–1.94; P = 0.02) which stated that the results were significant because P < 0.05 and the diamond did not touch the vertical line.

The results of the analysis on the cohort study design sub-group to see the sex comparison in COVID-19 patients with DM resulted in I ^2^ = 0% which indicated no heterogeneity between studies. Subtotal OR 5.71 (95%CI = 2.44–13.36; P < 0.0001) which indicates that the results were significant because P < 0.05 and the diamond did not touch the vertical line.

The results of the literature analysis to see the sex comparison between men and women in COVID-19 patients with DM overall yielded a value of I ^2^ = 59% which indicated heterogeneity between studies, so the random effects model was used. Total OR 3.28 (95% CI = 1.26–8.52; P = 0.01) with a confidence interval for the population between 1.26 to 8.52 (P = 0.01) indicated that the results were significant because P < 0.05. In addition, the diamond did not touch the vertical line, thus proving that male COVID-19 patients were more at risk of having DM than female patients.

### The Relationship between Prothrombin Time and Diabetes Mellitus in COVID-19 Patients

Fifteen literature included the distribution of prothrombin time values associated with the incidence of COVID-19 in DM and non-DM. Among them were 7 literature cohort research designs and 8 case-control literatures. Characteristics of prothrombin time in patients based on the study literature can be seen in Table 4 below.

**Table 4.** Study of prothrombin time (PT) values in diabetes mellitus (DM) patients with COVID-19 and non-diabetic patients with COVID-19 in Mean and Standard Deviation (SD).

Journal	Research design	NOS Score	PT value on DM (Mean ± SD)	PT value on non-DM (Mean ± SD)
Liu (c) *et al.* ^34^	Cohort	9	13.675 ± 0.325	13.55 ± 0.2
Elemam *et al.* ^8^	Cohort	9	12.45 ± 1.629	12.76 ± 6.622
Chen (a) *et al.* ^7^	Cohort	9	11.7 ± 0.333	11.3 ± 0.267
Chen (b) *et al.* ^22^	Cohort	9	11.525 ± 0.226	12.25 ± 0.3
Shang *et al.* ^44^	Cohort	9	12.73 ± 0.336	12.325 ± 0.283
Zhang (a) *et al.* ^51^	Cohort	9	12.73 ± 0.3	13.075 ± 0.25
Zhang *et al.* ^50^	Cohort	9	14.81 ± 0.707	13.686 ± 0.377
Akbariqomi *et al.* ^17^	Case-control	8	12.57 ± 0.183	12.475 ± 0.183
Bhandari *et al.* ^21^	Case-control	7	12.97 ± 0.662	12.63 ± 0.375
Dyusupova *et al.* ^27^	Case-control	8	13.71 ± 1.575	12.375 ± 0.416
Huang *et al.* ^10^	Case-control	8	11.65 ± 0.233	11.5 ± 1.667
Li *et al.* ^12^	Case-control	8	12.425 ± 0.283	12.225 ± 0.283
Liu (d) *et al.* ^35^	Case-control	8	11.425 ± 0.183	11.425 ± 0.15
Shi *et al.* ^45^	Case-control	8	12.15 ± 0.266	12.025 ± 0.216
Yan *et al.* ^18^	Case-control	8	14.86 ± 0.85	14.325 ± 0.383

NOS: Newcastle-Ottawa Scale.

Based on Table 4, the distribution of prothrombin time values in the incidence of COVID-19 with DM is compared with non-DM in the form of Median and IQR converted into the Mean and SD which has been converted in Table 4.

**Figure 4.**
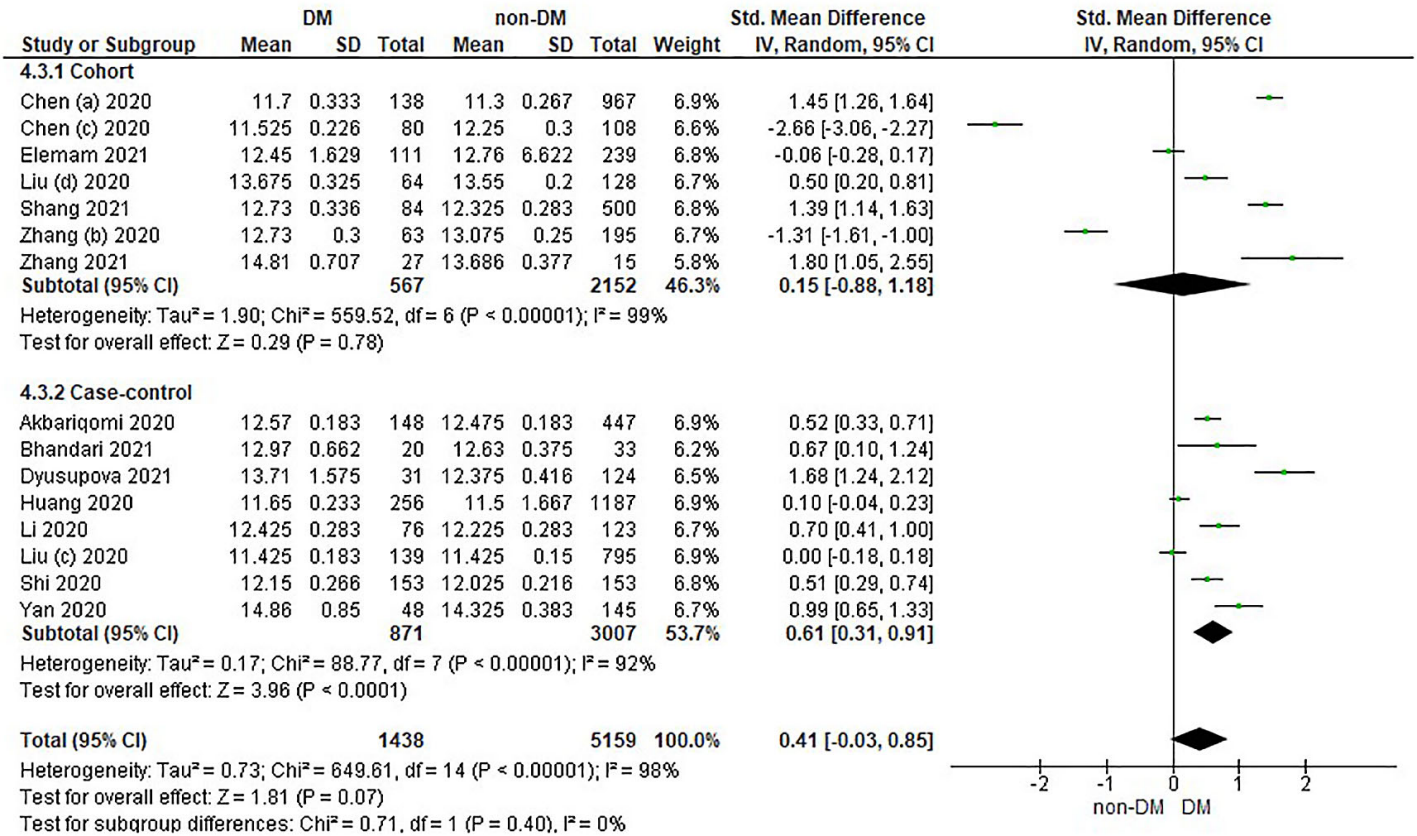
Forest plot of the relationship between prothrombin time values in diabetes mellitus patients with COVID-19 and non-diabetes mellitus with COVID-19.

Overall, it shows that the prothrombin time value in patients with DM has a slightly higher value compared to non-DM and as many as 3 studies have the opposite data. Forest plot analysis of the relationship between prothrombin time and the incidence of COVID with DM and non-DM can be seen in Figure 4.

The results of the literature analysis in the cohort study design subgroup to compare the prothrombin time values in COVID-19 patients with DM and non-DM resulted in I ^2^ = 99% which indicated heterogeneity between studies. Subtotal Standardized Mean Difference (SMD) 0.15 (95%CI = -0.88–1.18; P = 0.78) which means that the result was not significant because P > 0.05 and the diamond touched the vertical line.

Results of literature analysis in the case-control study design sub-group to compare the prothrombin time values in COVID-19 patients with DM and non-DM produced I ^2^ = 92% which indicated heterogeneity between studies. Subtotal Standardized Mean Difference (SMD) 0.61 (95%CI = 0.31–0.91; P < 0.0001) which means that the result is significant because P < 0.05 and the diamond did not touch the vertical line.

The results of the literature analysis to see the comparison of the prothrombin time value in COVID-19 patients with DM and COVID-19 patients without a history of DM overall yielded a value of I ^2^ = 98% which indicated heterogeneity between studies, so the random effects model was used. Total Standardized Mean Difference (SMD) 0.41 (95%CI = -0.03–0.85; P = 0.07) with a confidence interval for the population between -0.03 to 0.85 (P = 0.07) showed that there were insignificant results because P < 0.05. In addition, the diamond touched the vertical line thus proving that the prothrombin time value was the same in both COVID-19 patients with DM and COVID-19 patients who did not have comorbid DM before being hospitalized.

## Discussion

This systematic review and meta-analysis included 46 articles with a total number of 1,325,334 patients who were positive for COVID-19 and divided into diabetic and non-diabetic groups which were analyzed for age, sex and prothrombin time values.

Diabetes is reported to be one of the comorbidities that increases the progression and mortality of COVID-19. Diabetes can be a risk factor because of the increase in serum ACE2 in diabetic patients. In addition, patients taking inhibitors of angiotensin-converting enzyme (ACEIs) and angiotensin II receptor blockers (ARBs) showed overexpression of ACE2, the COVID-19 entry receptor. ^8^

The results of a systematic study and meta-analysis on the age variable, showed that patients with COVID-19 with DM were significantly older than non-diabetic patients. There is a correlation between age and the innate immune system as has been reviewed elsewhere and concluded that the elderly are particularly susceptible to developing more infections because the innate immune system declines gradually with older age. ^9^

The relationship between age and the incidence of COVID-19 in the DM group compared to non-DM is in line with several research results which state that patients infected with COVID-19 with comorbid diabetes are older than non-diabetics. In both patients with or without diabetes the severity of the disease increases with age. ^10^ Another study also found that diabetic patients were significantly older and had more severe symptoms than non-diabetic patients, ^11^ the COVID-19 patients with diabetes had a higher age than non-diabetics, ^12^ COVID-19 patients with pre-existing diabetes were older than those without. ^7^ Another study stated that diabetic and non-diabetic population significantly different in age but a slightly older non-diabetic population. ^13^

The results of the study on the gender variable, showed that men were more at risk of exposure to the disease and had more severe symptoms than women. Gender differences affect clinical outcome and prognosis, with males at higher risk than females. Male patients may express higher ACE2 which is regulated by male sex hormones. ^9^

The relationship between sex and the incidence of COVID-19 in the DM group is in line with several research results, such as having a much larger male population than female, ^14^ twice as many male patient subjects as confirmed positive for COVID-198, the presentation of diabetic men at high risk of mortality and the number of hospitalizations is higher in diabetic men than women and in other comorbid diseases. ^15^ In contrast to a study, in the data there were more female patients than men, although there were more men in the diabetes group than non-diabetics but in both groups had more female patients. ^16^

The prothrombin time variable showed the same prothrombin time value in both diabetic and non-diabetic patients. Theoretically, COVID-19 patients with DM have a prolonged prothrombin time value, as well as the results in the case-control study design sub-group as seen in Figure 3 which shows a difference, namely a prolonged prothrombin time value in the DM group. Diabetic patients in a prothrombotic state due to hyperglycemia and chronic hyperinsulinism make all phases of coagulation abnormal. ^7^ Non-survivors have a prolonged prothrombin time compared to survivors. The timing of increases in D-dimer, prothrombin time, and activated partial thromboplastin time, with decreased fibrinogen and platelet counts, also coincided with the duration of hospitalization, ranging from 7 to 10 days after admission. Patients who are still hospitalized or have good prognostic factors are likely to have non-prolonging prothrombin time. ^54^

The relationship between prothrombin time and the incidence of COVID-19 in the DM and non-DM groups is in line with several studies. ^17^ The prothrombin time values in both groups were relatively the same and did not prolong. ^12^ The prothrombin time values were almost the same in both groups and within the normal range. ^10^ In contrast to a study that showed a slight difference in the prothrombin time value in the diabetic group, which was prolonged compared to the non-diabetic group, which was still within normal limits. ^18^

This study has research limitations, there are only a few studies on COVID-19 with DM as the outbreak only occurred at the end of 2019. Research on COVID-19 with DM is relatively new, and it needs to be studied further. This meta-analysis also does not study the relationship between the onset and severity of COVID-19 with diabetes.

## Conclusion

COVID-19 patients with older age are at a higher risk of contracting DM compared to younger COVID-19 patients. Likewise, male COVID-19 patients are more susceptible to DM compared to female COVID-19 patients. As DM is a comorbidity in COVID-19, it can be concluded that old age and male sex are associated with a more severe disease. On the other hand, the prothrombin time values in both diabetic and non-diabetic groups tended to be the same and within normal limits.

### Suggestion

Researchers are expected to conduct further studies on the relationship between age and gender in COVID-19 patients with DM, so that the data obtained from the results of this meta-analysis are more relevant when applied in Indonesia.

Clinicians are expected to provide health care, especially for patients with DM who are old and male in the era of the COVID-19 pandemic to reduce the risk factors for severity and mortality of diabetic patients being infected with COVID-19.

Researchers are expected to conduct further studies on prothrombin time in COVID-19 patients with DM for a more detailed understanding.

## Data availability

### Underlying data

All data underlying the results are available as part of the article and no additional source data are required.

### Reporting guidelines

Figshare: PRISMA checklist for ‘The relationship of age, sex and prothrombin time related to the severity and mortality of COVID-19 patients with diabetes mellitus: a systematic review and meta analysis’. https://doi.org/10.6084/m9.figshare.18865103. ^56^

Data are available under the terms of the Creative Commons Attribution 4.0 International license (CC-BY 4.0).

## Author contributions

**Audrey Fabianisa Mirza:** Conceptualization, formal analysis, methodology, investigation, visualization, writing – original draft preparation, writing – review & editing.

**Ceria Halim:** Formal analysis, writing – original draft preparation, writing – review & editing.

**Mutiara Indah Sari:** Conceptualization, formal analysis, methodology, project administration, supervision, funding acquisition.
