# Supplementary material for: The relationship of age, sex and prothrombin time related to the severity of COVID-19 patients with diabetes mellitus: a systematic review and meta analysis
Source: F1000Res. 2025 Oct 14;11:729. Originally published 2022 Jul 1. [Version 7] doi: 10.12688/f1000research.107398.7 (PMC11889402; doi:10.12688/f1000research.107398.7)
Supplement: Peer review history

# Reviewer report: Reviewer response for version 6

**DOI:** 10.5256/f1000research.169997.r368187

**Published:** 2025-03-07

**Authors:** Chenxiao Wang

**Affiliations:** 1 Tulane University, New Orleans, USA

The manuscript systematically reviews and conducts a meta-analysis to investigate how age, sex, and prothrombin time (PT) values relate to COVID-19 severity among patients with diabetes mellitus (DM). The authors analyzed data from 45 studies sourced from prominent databases, applying robust inclusion and exclusion criteria. Overall, the study concludes that older age and male gender significantly increase COVID-19 severity risk among patients with DM, whereas prothrombin time values were slightly prolonged in DM patients compared to non-DM patients, though not significantly so overall.

 The manuscript addresses a clinical issue, and the authors addressed all the questions reviewers raised.

Are the rationale for, and objectives of, the Systematic Review clearly stated?

Yes

Is the statistical analysis and its interpretation appropriate?

Yes

If this is a Living Systematic Review, is the ‘living’ method appropriate and is the search schedule clearly defined and justified? (‘Living Systematic Review’ or a variation of this term should be included in the title.)

Not applicable

Are sufficient details of the methods and analysis provided to allow replication by others?

Yes

Are the conclusions drawn adequately supported by the results presented in the review?

Yes

Reviewer Expertise:

COVID-19

I confirm that I have read this submission and believe that I have an appropriate level of expertise to confirm that it is of an acceptable scientific standard.

# Reviewer report: Reviewer response for version 6

**DOI:** 10.5256/f1000research.169997.r313402

**Published:** 2024-08-27

**Authors:** Ipsa Arora

**Affiliations:** 1 Endocrinology, Central Maine Medical Center, Lewiston, Maine, USA

Mirza et al. conducted a systematic review and meta-analysis to examine the relationship between age, gender, and prothrombin time in COVID-19 patients requiring hospitalization, with or without diabetes as a comorbidity. They found that hospitalized COVID-19 patients with diabetes were generally older and predominantly male. Although a statistically significant association with prothrombin time was not identified, hospitalized patients with both COVID-19 and diabetes tended to have higher prothrombin time values.

 I recommend the following clarifications to enhance the clarity and usefulness of this publication for the readers of this journal.

1. **Abstract**: The study's aim is not clearly articulated in the abstract.
2. **Conclusion**: The statement "patients with COVID-19 who have diabetes have a higher risk as compared to those without diabetes" is vague. Please specify the higher risk of what—e.g., higher risk of hospitalization, severe outcomes, or mortality.
3. **Introduction**: In the last sentence where the study's aim is mentioned, there seems to be a discrepancy. The study does not investigate the relationship between age, gender, and prothrombin time with the "severity of COVID-19." Instead, it examines these parameters in relation to COVID-19 cases requiring hospitalization. This should be clarified, especially since the limitations (under discussion) indicate that the meta-analysis does not explore the relationship with COVID-19 severity.
4. **Methods**: In the literature search section (2019-2021), it is recommended to specify the exact months for each year.
5. **Inclusion Criteria**: The first line states, "all retrospective studies... had data on patients' age, sex, and prothrombin time." It may be more accurate to say "age, sex, or prothrombin time," as not all 45 studies included data on all three variables.
6. **Page 7/25**: In the section discussing the relationship between age and diabetes in COVID-19 patients, "incidence" might not be the most appropriate term; "prevalence" could be more accurate. The same applies to page 9/25 regarding the relationship between sex and diabetes in COVID-19 patients.
7. **Page 10/25**: The phrase "Based on Table 3..." does not appropriately represent the study’s findings. It should be revised to indicate that "male patients with diabetes have a higher risk of hospitalization compared to female patients with diabetes."
8. **Page 10/25**: In the section discussing the relationship between prothrombin time and diabetes in COVID-19 patients, consider revising the conclusion to state that "no statistically significant difference was identified in prothrombin time between hospitalized COVID-19 patients with or without diabetes; however, values were generally higher in patients with diabetes."
9. **Page 12/25**: The last line of the results section ("The SMD >0... more severe illness") is unclear and may need to be rewritten, taking into account the points raised in comment 8.
10. **Discussion, Paragraph 3**: Please specify that the results discussed are applicable only to hospitalized patients.
11. **Discussion, Paragraph 4**: The term "incidence" is used incorrectly; please refer to comment 6 for guidance.
12. **Discussion, Paragraph 5**: The first statement may be inaccurate and should be revised based on the information discussed in comment 7.
13. **Discussion, Paragraph 7**: The conclusion should specify that it pertains to patients with COVID-19 requiring hospitalization.
14. **Discussion, Paragraph 8**: The mention of "incidence" should be corrected as indicated in comment 6.

Are the rationale for, and objectives of, the Systematic Review clearly stated?

Partly

Is the statistical analysis and its interpretation appropriate?

I cannot comment. A qualified statistician is required.

If this is a Living Systematic Review, is the ‘living’ method appropriate and is the search schedule clearly defined and justified? (‘Living Systematic Review’ or a variation of this term should be included in the title.)

Not applicable

Are sufficient details of the methods and analysis provided to allow replication by others?

Yes

Are the conclusions drawn adequately supported by the results presented in the review?

Partly

Reviewer Expertise:

Endocrinology

I confirm that I have read this submission and believe that I have an appropriate level of expertise to confirm that it is of an acceptable scientific standard, however I have significant reservations, as outlined above.

# Reviewer report: Reviewer response for version 6

**DOI:** 10.5256/f1000research.169997.r312342

**Published:** 2024-08-16

**Authors:** Fajri Marindra Siregar

**Affiliations:** 1 Universitas Riau, Pekanbaru, Riau, Indonesia

The author has revised the manuscript according to the reviewer's input.

Are the rationale for, and objectives of, the Systematic Review clearly stated?

Yes

Is the statistical analysis and its interpretation appropriate?

I cannot comment. A qualified statistician is required.

If this is a Living Systematic Review, is the ‘living’ method appropriate and is the search schedule clearly defined and justified? (‘Living Systematic Review’ or a variation of this term should be included in the title.)

No

Are sufficient details of the methods and analysis provided to allow replication by others?

Partly

Are the conclusions drawn adequately supported by the results presented in the review?

No

Reviewer Expertise:

biochemistry, health and medicine

I confirm that I have read this submission and believe that I have an appropriate level of expertise to confirm that it is of an acceptable scientific standard.

# Reviewer report: Reviewer response for version 5

**DOI:** 10.5256/f1000research.169449.r306939

**Published:** 2024-08-01

**Authors:** Fajri Marindra Siregar

**Affiliations:** 1 Universitas Riau, Pekanbaru, Riau, Indonesia

1) There is still a difference between the number of articles that entered the final stage in the abstract and results sections

 2) The conclusion in the abstract still states "As diabetes is a comorbidity in COVID-19, it can be concluded that old age and male sex are associated with a more severe disease." this cannot be answered based on the results of the study presented.

 Authors are advised to recheck the manuscript before submitting a revision, and ensure that what is written in the abstract is in line with the data presented in the contents of the manuscript.

Are the rationale for, and objectives of, the Systematic Review clearly stated?

Yes

Is the statistical analysis and its interpretation appropriate?

I cannot comment. A qualified statistician is required.

If this is a Living Systematic Review, is the ‘living’ method appropriate and is the search schedule clearly defined and justified? (‘Living Systematic Review’ or a variation of this term should be included in the title.)

No

Are sufficient details of the methods and analysis provided to allow replication by others?

Partly

Are the conclusions drawn adequately supported by the results presented in the review?

No

Reviewer Expertise:

biochemistry, health and medicine

I confirm that I have read this submission and believe that I have an appropriate level of expertise to confirm that it is of an acceptable scientific standard, however I have significant reservations, as outlined above.

# Response

**Published:** 2024-08-03

**Authors:** Mutiara Indah Sari

**Affiliations:** Universitas Sumatera Utara, Indonesia

Thank you for the review

**1:** There is still a difference between the number of articles that entered the final stage in the abstract and results sections

**2:** The conclusion in the abstract still states "As diabetes is a comorbidity in COVID-19, it can be concluded that old age and male sex are associated with a more severe disease." this cannot be answered based on the results of the study presented.

**Response:** I apologize for any inconsistencies. I have reviewed the manuscript and ensured that it is revised before, I revised it again to match the abstract with the data content.

 Sincerely,

 Mutiara Indah Sari

# Reviewer report: Reviewer response for version 4

**DOI:** 10.5256/f1000research.168875.r301754

**Published:** 2024-07-19

**Authors:** Fajri Marindra Siregar

**Affiliations:** 1 Universitas Riau, Pekanbaru, Riau, Indonesia

The revision submitted by the author has not addressed the reviewer's questions.

 1) The author should clarify the criteria used to evaluate each variable in the article, considering the varying number of assessed articles. According to Figure 1, there are a total of 46 articles. However, out of these, only 31 articles are used for the age variable, 5 articles are used for gender, and 15 articles are used for Prothrombin Time. For every variable, why not use all of the articles in the final stage? Further clarification is required. 

 Apart from that, clarification is needed due to the inconsistency in the number of articles at the final stage, whether 46 or 45 because there is a difference between the abstract and the results section.

 2) In the conclusion the author states "Since DM is a comorbidity in COVID-19, it can be concluded that older age and male sex are associated with more severe disease". While this proposition may be true in theory, the findings of this study do not address this particular aspect. It is advisable to omit that particular sentence from the conclusion.

Are the rationale for, and objectives of, the Systematic Review clearly stated?

Yes

Is the statistical analysis and its interpretation appropriate?

I cannot comment. A qualified statistician is required.

If this is a Living Systematic Review, is the ‘living’ method appropriate and is the search schedule clearly defined and justified? (‘Living Systematic Review’ or a variation of this term should be included in the title.)

No

Are sufficient details of the methods and analysis provided to allow replication by others?

Partly

Are the conclusions drawn adequately supported by the results presented in the review?

No

Reviewer Expertise:

biochemistry, health and medicine

I confirm that I have read this submission and believe that I have an appropriate level of expertise to confirm that it is of an acceptable scientific standard, however I have significant reservations, as outlined above.

# Response

**Published:** 2024-07-20

**Authors:** Mutiara Indah Sari

**Affiliations:** Universitas Sumatera Utara, Indonesia

Thank you for the review.

**1:** The author should clarify the criteria used to evaluate each variable in the article, considering the varying number of assessed articles. According to Figure 1, there are a total of 46 articles. However, out of these, only 31 articles are used for the age variable, 5 articles are used for gender, and 15 articles are used for Prothrombin Time. For every variable, why not use all of the articles in the final stage? Further clarification is required. 

 Apart from that, clarification is needed due to the inconsistency in the number of articles at the final stage, whether 46 or 45 because there is a difference between the abstract and the results section.

**Response:** The literature used is 45. We apologize for the discrepancy. We have thoroughly reviewed the literature used to process 45 data. There were duplicated references in Table 1 earlier, however, this does not affect the results of the forest plot data processing, only the numerical counts in the characteristics.

 We cannot use all literature for each variable because not all data for each variable is available in every literature we used. Therefore, in total for the three variables, we used 45 literature sources, with 31 articles used for age, 5 articles for gender, and 15 articles for prothrombin time. Among these, there are overlapping articles that contain data for two variables.​​​​​​​

**2:** In the conclusion the author states "Since DM is a comorbidity in COVID-19, it can be concluded that older age and male sex are associated with more severe disease". While this proposition may be true in theory, the findings of this study do not address this particular aspect. It is advisable to omit that particular sentence from the conclusion.

**Response:** We have omit that particular sentence from the conclusion. 

 Sincerely,

 Mutiara Indah Sari

# Reviewer report: Reviewer response for version 3

**DOI:** 10.5256/f1000research.168129.r294009

**Published:** 2024-07-05

**Authors:** Fajri Marindra Siregar

**Affiliations:** 1 Universitas Riau, Pekanbaru, Riau, Indonesia

The author has made several improvements, but the following 2 things still concern reviewers:

 1) The author should clarify the criteria used to evaluate each variable in the article, considering the varying number of assessed articles. According to Figure 1, there are a total of 46 articles. However, out of these, only 31 articles are used for the age variable, 5 articles are used for gender, and 15 articles are used for Prothrombin Time. Further clarification is required.

 In the latest revision the author only mentions "The final results after selection got a total of 46 articles that were included in this meta-analysis study. Within these, 31 articles are utilized for age, 5 articles are utilized for gender, and 15 articles are utilized for prothrombin time." without any further explanation.

 2) In the conclusion the author states "Since DM is a comorbidity in COVID-19, it can be concluded that older age and male sex are associated with more severe disease". While this proposition may be true in theory, the findings of this study do not address this particular aspect.

Are the rationale for, and objectives of, the Systematic Review clearly stated?

Yes

Is the statistical analysis and its interpretation appropriate?

I cannot comment. A qualified statistician is required.

If this is a Living Systematic Review, is the ‘living’ method appropriate and is the search schedule clearly defined and justified? (‘Living Systematic Review’ or a variation of this term should be included in the title.)

No

Are sufficient details of the methods and analysis provided to allow replication by others?

Partly

Are the conclusions drawn adequately supported by the results presented in the review?

No

Reviewer Expertise:

biochemistry, health and medicine

I confirm that I have read this submission and believe that I have an appropriate level of expertise to confirm that it is of an acceptable scientific standard, however I have significant reservations, as outlined above.

# Response

**Published:** 2024-07-07

**Authors:** Mutiara Indah Sari

**Affiliations:** Universitas Sumatera Utara, Indonesia

Thank you for the review. We will revise the manuscript based on your suggestions.

**1:** In the latest revision the author only mentions "The final results after selection got a total of 46 articles that were included in this meta-analysis study. Within these, 31 articles are utilized for age, 5 articles are utilized for gender, and 15 articles are utilized for prothrombin time." without any further explanation.

**Response:** For further explanation, we have added a venn diagram included studies for each variables.

**2:** In the conclusion the author states "Since DM is a comorbidity in COVID-19, it can be concluded that older age and male sex are associated with more severe disease". While this proposition may be true in theory, the findings of this study do not address this particular aspect.

**Response:** We have added some explanation related to our study in the results section. All samples in the articles we reviewed were hospitalized patients, where the severity was more severe than those who were only isolated at home. Among patients with severe disease, we compared diabetic and non-diabetic group.

 Sincerely,

 Mutiara Indah Sari

# Reviewer report: Reviewer response for version 2

**DOI:** 10.5256/f1000research.167093.r286853

**Published:** 2024-06-18

**Authors:** Fajri Marindra Siregar

**Affiliations:** 1 Universitas Riau, Pekanbaru, Riau, Indonesia

The author has made several improvements to complete the information, especially in the methods section, but several things still need to be clarified.

1. The author should clarify the criteria used to evaluate each variable in the article, considering the varying number of assessed articles. According to Figure 1, there are a total of 46 articles. However, out of these, only 31 articles are utilized for the age variable, 5 articles are utilized for gender, and 15 articles are used for Prothrombin Time. Further clarification is required.
2. In the results section, the author compares the average age and prothrombin time between COVID-19 patients with and without DM. However, regarding the gender variable, the author only examines the comparison of male and female genders in COVID-19 patients with DM. To ensure consistency with the age and PT variables, it would be appropriate to analyze the sex ratio in COVID-19 patients with and without DM for the gender variable.
3. In the results section, the sentence "Total Standardised Mean Difference (SMD) 0.41 (95%CI = -0.03–0.85; P = 0.07) with a confidence interval for the population between -0.03 to 0.85 (P = 0.07) showed that there were insignificant results because P < 0.05." should be corrected to "Total Standardised Mean Difference (SMD) 0.41 (95%CI = -0.03–0.85; P = 0.07) with a confidence interval for the population between -0.03 to 0.85 (P = 0.07) showed that there were insignificant results **because P > 0.05.**"
4. The conclusion section has not adequately addressed the concerns raised by the previous reviewer. I suggest revising the conclusion to align it with the results section. Specifically, the revised conclusion should state that the findings of this meta-analysis indicate that COVID-19 patients with comorbid DM tend to be older and male compared to COVID-19 patients without DM. However, there was no significant difference in the results of prothrombin time.

Are the rationale for, and objectives of, the Systematic Review clearly stated?

Yes

Is the statistical analysis and its interpretation appropriate?

I cannot comment. A qualified statistician is required.

If this is a Living Systematic Review, is the ‘living’ method appropriate and is the search schedule clearly defined and justified? (‘Living Systematic Review’ or a variation of this term should be included in the title.)

No

Are sufficient details of the methods and analysis provided to allow replication by others?

Partly

Are the conclusions drawn adequately supported by the results presented in the review?

No

Reviewer Expertise:

biochemistry, health and medicine

I confirm that I have read this submission and believe that I have an appropriate level of expertise to confirm that it is of an acceptable scientific standard, however I have significant reservations, as outlined above.

# Response

**Published:** 2024-06-19

**Authors:** Mutiara Indah Sari

**Affiliations:** Universitas Sumatera Utara, Indonesia

Dear Reviewer,

 Thank you for the review. We will revise the manuscript based on your suggestions.

**1:** The author should clarify the criteria used to evaluate each variable in the article, considering the varying number of assessed articles. According to Figure 1, there are a total of 46 articles. However, out of these, only 31 articles are utilized for the age variable, 5 articles are utilized for gender, and 15 articles are used for Prothrombin Time. Further clarification is required.

**Response:** Further clarification is added as suggested. 

**2:** In the results section, the author compares the average age and prothrombin time between COVID-19 patients with and without DM. However, regarding the gender variable, the author only examines the comparison of male and female genders in COVID-19 patients with DM. To ensure consistency with the age and PT variables, it would be appropriate to analyze the sex ratio in COVID-19 patients with and without DM for the gender variable.​​​​​​​

**Response:** We analyzed gender differences between male and female to assess gender as a risk factor, particularly among COVID-19 patients with comorbid diabetes, to determine whether more male or female were hospitalized. We processed gender data using odds ratios, unlike age and prothrombin time variables, which utilized mean and standard deviation. Additionally, there was a lack of odds ratio data for non-diabetic patients in the literature we reviewed.  ​​​​​​​

**3:** In the results section, the sentence "Total Standardised Mean Difference (SMD) 0.41 (95%CI = -0.03–0.85; P = 0.07) with a confidence interval for the population between -0.03 to 0.85 (P = 0.07) showed that there were insignificant results because P < 0.05." should be corrected to "Total Standardised Mean Difference (SMD) 0.41 (95%CI = -0.03–0.85; P = 0.07) with a confidence interval for the population between -0.03 to 0.85 (P = 0.07) showed that there were insignificant results **because P > 0.05.**"​​​​​​​

**Response:** We will rectify the typographical error.

**4:** The conclusion section has not adequately addressed the concerns raised by the previous reviewer. I suggest revising the conclusion to align it with the results section. Specifically, the revised conclusion should state that the findings of this meta-analysis indicate that COVID-19 patients with comorbid DM tend to be older and male compared to COVID-19 patients without DM. However, there was no significant difference in the results of prothrombin time.

**Response:** We will revise the conclusion to align it with the results.

 Sincerely,

 Mutiara Indah Sari

# Reviewer report: Reviewer response for version 1

**DOI:** 10.5256/f1000research.118616.r269067

**Published:** 2024-05-16

**Authors:** Fajri Marindra Siregar

**Affiliations:** 1 Universitas Riau, Pekanbaru, Riau, Indonesia

The author should properly clarify the search method utilised in each database, as each database has a unique search algorithm, so that other scholars can easily adapt it.

 Next, the author must certify that the results of the literature search are articles published until when?

 The analysis of statistical results states that there is very high (significant) variability in each variable; has the author made any efforts to lessen this heterogeneity? (Please refer to https://handbook-5-1.cochrane.org/chapter_9/9_5_3_strategies_for_addressing_heterogeneity.htm)

 Furthermore, the conclusions presented are not in accordance with the results obtained by the author. In the conclusion the author stated "Patients with older age with diabetes tend to have more severe disease than non-diabetics" even though the meta-analysis carried out only compared age between the Covid group with DM vs without DM, there was no study of morbidity variables.

 Likewise, the statement "Patients with diabetes who are hospitalized are more likely to be male, indicating that males are more susceptible to severe disease" in our opinion is not appropriate considering that there are no variables for hospitalization status or degree of disease that were studied by the author in the meta-analysis section.

 Furthermore, considering that there are still several limitations that may not be avoidable, these need to be explored further by the author in the discussion section, such as the influence of disease onset and disease degree.

Are the rationale for, and objectives of, the Systematic Review clearly stated?

Yes

Is the statistical analysis and its interpretation appropriate?

I cannot comment. A qualified statistician is required.

If this is a Living Systematic Review, is the ‘living’ method appropriate and is the search schedule clearly defined and justified? (‘Living Systematic Review’ or a variation of this term should be included in the title.)

No

Are sufficient details of the methods and analysis provided to allow replication by others?

Partly

Are the conclusions drawn adequately supported by the results presented in the review?

No

Reviewer Expertise:

biochemistry, health and medicine

I confirm that I have read this submission and believe that I have an appropriate level of expertise to confirm that it is of an acceptable scientific standard, however I have significant reservations, as outlined above.

# Response

**Published:** 2024-05-27

**Authors:** Mutiara Indah Sari

**Affiliations:** Universitas Sumatera Utara, Indonesia

Dear Reviewer,

 Thank you for the review. We will revise the manuscript based on your suggestions.

**1:** The author should properly clarify the search method utilised in each database, as each database has a unique search algorithm, so that other scholars can easily adapt it.

**Response:** We will add more details on the search strategy such as the filters for each database in the text.

**2:** Next, the author must certify that the results of the literature search are articles published until when?

**Response:** We will add the publication years for the included literatures in the text.

**3:** The analysis of statistical results states that there is very high (significant) variability in each variable; has the author made any efforts to lessen this heterogeneity?

**Response:** Referring to the handbook, we performed point 1 (rechecking the data) and 7 (studies exclusion) to reduce heterogeneity. We will add brief information on this in the text.

**4:** Furthermore, the conclusions presented are not in accordance with the results obtained by the author. In the conclusion the author stated "Patients with older age with diabetes tend to have more severe disease than non-diabetics" even though the meta-analysis carried out only compared age between the Covid group with DM vs without DM, there was no study of morbidity variables.

 Likewise, the statement "Patients with diabetes who are hospitalized are more likely to be male, indicating that males are more susceptible to severe disease" in our opinion is not appropriate considering that there are no variables for hospitalization status or degree of disease that were studied by the author in the meta-analysis section.

**Response:** It is true that our result is comparing COVID patients with vs without DM, so we will make changes on the conclusion and a few other parts of the text (for consistency and clarification) based on your suggestion. However, we would like to note that as DM is a comorbidity in COVID, we use it as an indicator of a more severe disease.

**5:** Furthermore, considering that there are still several limitations that may not be avoidable, these need to be explored further by the author in the discussion section, such as the influence of disease onset and disease degree.

**Response:** We will add the limitations to the text as suggested.

 Sincerely,

 Mutiara Indah Sari
